# The city emptied and the homes and hospitals turned into 'the world'. A sociological approach

**DOI:** 10.12688/f1000research.52097.1

**Published:** 2021-05-27

**Authors:** Juan Coca, Juan A. Roche Cárcel

**Affiliations:** 1 Social Research Unit in Health and Rare Diseases, University of Valladolid, Soria, 42004, Spain; 2 Department of Sociology I, University of Alicante, Alicante, 03080, Spain

**Keywords:** Frame visual, social enclosure, cities emptied, hospitals, houses, social paradox, COVID-19, social interrelations.

## Abstract

Background: The coronavirus pandemic has generated social measures in order to contend virus expansion, and deaths. One of the most important political norms in the first wave was the domestic enclosure. This measure generates social, psychological and personal problems. Objective: The aim of this paper is to analyze the social impact of this home confinement through the study of journalistic images. Methods: We use a set of images selected previously according our epistemic necessities. Results: The results show that the fundamental elements of the current life were questioned. In fact, social space, and the own society had collapsed. Also, the enclosure of Spanish populations has been accompanied by the intensification of the individualism, but also has generated an increase of the ideal of communitas. Conclusions: Coronavirus enclosure has produced that the cities have emptied, and the houses and hospitals, were the last refuge of society. This phenomenon has generated that population has been accompanied by the paradox intensification of social relationships.

## Introduction

The dramatic situation faced by Spain as one of the countries most strongly hit by COVID-19 (regarding both the number of deaths and that of variants of the virus) led the Spanish government to declare a “state of emergency,” a legal and constitutional figure meant to suppress multiple rights of citizens in order to preserve their health and safety. This remained in force between March 14th and May 18th 2020 during the first phase. In accordance with our starting hypothesis that will be eventually confirmed or rejected on the following pages, the aforesaid state of emergency has important consequences in at least three respects. Firstly, cities —amongst them Alicante— became deserted, which in turn meant that due to the complete absence of action and social relationships, the lack of a political agora, and the forced social distancing between bodies together with their confinement inside homes, society was somehow emptied too. Secondly, as a result of the above, both homes and hospitals have expanded their traditional functions and transformed into a real “world” where the usual activities as well as new ones are carried out. In this confinement situation, the population perceives hospitals as the only ones with capacity to save people. For this reason, the traditional activity of hospitals is collectively perceived as more relevant, more “real” and closer to the community. And, thirdly, such a transformation has gone hand in hand with a strengthening of individualism and, paradoxically, of the *communitas* too. We know that individualization has played an important role in both classical and modern sociology. Indeed, sociological theories start from the importance of people’s choice. Faced with social reality, individuals can make the choice to separate themselves or to draw closer to others. In other words, both characteristics of the Society of Individualization ^1- 7^ and others typically associated with the Society of Separativity ^8^ and the *communitas* ^9^ have been simultaneously reinforced.

## Methods

### Visual Sociology and Methodology: the hermeneutics and association of the iconological analysis, the documentary method, and the visual frame

Our implementation of the starting hypothesis described above will rely not only on two perspectives of sociology but also on two basic methods aimed at analyzing the content of the photographs taken by the Alicante-born photojournalist Rafa Arjones, which will be discussed in this research. The first key reference for our work - Weberian Comprehensive or Interpretative Sociology, ^10, 11^ according to which both the social world and the relationships that it generates are meaningful — was chosen in the hope that, through “correspondence in meaning” or “elective affinities,” it could help us identify the common links between the different cognitive dimensions —aesthetic, ethical, economic, political, religious, and social— that modernity has fragmented and, more specifically, the physical and socially constructed reality inside which Alicante developed throughout the COVID-19 lockdown. The second main topic behind our approach can be found in Visual Sociology, which mainly focuses on two issues: analyzing the image in its various forms; and using this analysis to collect data about social reality. Both dimensions converge on the ultimate goal of this subdiscipline, namely, the production of social knowledge through images. ^12^ It should be noted, though, that Social Sciences have historically tended to privilege written sources, thus marginalizing or even excluding others such as sound and visual sources. More precisely, the latter “have been rejected either as mere ‘data’ or as simple ‘research instruments’”. ^13^ Notwithstanding the above, Visual Sociology takes advantage of certain excellent sociological antecedents. ^14- 17^ Furthermore, as stated by Eduardo Bericat, ^18^ numerous studies have revolved around the Sociology of Photography. Examples close to our research project include the works of Bruce Jackson —life in prison (1977, 1978)—; Douglas Harper —the vagabonds (1978)—; Lewis Wickes Hine —a sociologist and photographer who called his works “photointerpretations” (from working children in the United States (1908))—; and John Grady —the segregation of the black race (2007)—. ^13, 18^ This subdiscipline is currently experiencing a new rebirth, with researchers more experienced in new technologies. ^10^ This sociological tradition which links photography and sociology —both of them modalities to explore the social — has included, on the one hand, three perspectives when reading any photograph (according to H. Becker): journalistic; documentary; and Visual-Sociology-inspired. ^19^ On the other hand, two great approaches have emerged in Visual Sociology: working with or on already-taken photographs. The first case refers to the incorporation of that practice by the actual research team, either working from the photographs or creating them. Photography thus plays a relevant role in the research process itself, albeit changing its consideration: as a secondary source (insofar as the purposes sought with the photographs used had nothing to do with the research process, e.g. giving rise to a collection, a fund or an archive); or as a primary source, because the former are original observations made for or through relevant research studies. ^13, 20^ This article belongs within the sociological work on photojournalistic images, since it discusses work on photographs from a documentary repertoire, in this case of a professional nature, as is the case with the Spanish press. In any case, the photographs under analysis raise a special sociological interest when regarded in terms of self-presentation and approached on the basis of the actual conception that a society has of itself at a given moment, or expressed differently, of how that society imagines itself. ^21^ More specifically, these images were taken — as seen below — by the photojournalist Rafa Arjones, while the state of emergency remained in force (during stage 0). Finally, the following additional considerations about photography should be borne in mind:

1. Photography and Sociology have contemporaneity in common; ^22^ 2. Photographic images are “index” i.e. a trace verifying the existence of real phenomena for which these images are references; ^23^ 3. The emotional component of photography always outweighs that of any written text and consequently helps humanize social problems that lonely and uprooted people go through. ^24^ In other words, a photograph allows its viewers to see those underprivileged persons as individual human beings who suffer and fight and no longer as impersonal generalized abstractions; 4. The interpretation of photographic images cannot cease to be historical, insofar as it depends on the prior knowledge about the situation and considering that several years elapse before the analysis ^25^ and the publication of dozens of works; 5. Photography provides an alternative discourse on the pandemic experience, on this occasion from the perspective of an experienced photojournalist; ^26^ 6. As a tool for social analysis, photography largely helps in the construction of social reality; ^27^ 7. Since photojournalists are the ones who have to produce clear, self-explanatory and descriptive images than can appear on the pages of media, ^19^ their photographs become a first-rate social document. Photojournalists additionally serve as an essential source of information through which the extreme situations undergone by human beings during the lockdown can be given an affective touch.

The search for meaning leads us to perform a qualitative or content analysis ^28, 29^ materialized through heuristics or hermeneutics and the combination of three visual methods. The “heuristic or interpretive method” comes from social hermeneutics, a particularly useful science when it comes to comprehensive sociology, insofar as interpretation constitutes its central problem. ^30^ Certainly, what social hermeneutics helps interpret is the things themselves —though seen in their own context: ^31^ with the aim of finding the deep keys to photographic images, or expressed differently, of revealing their inner meaning from the external ideological discourse. ^32^ Ideology is a system of ideas, values, and precepts which organize or legitimize the actions of individuals or groups, whereas discourse can be described as an action and social interaction mode located in social contexts, i.e. both discourse itself and its mental dimensions (e.g. its meanings) form part of specific situations and social structures. ^33^ The three audiovisual methods involved here are: Panofsky’s “iconological —or iconographic— analysis”, ^34, 35^ which in fact has a long sociological tradition; and K. Mannheim’s “documentary method”, ^36^ which deals with the social information supplied by images; and “visual framing,” which visually situates reality and contextualizes information. ^25, 29, 37^ In short, this connection facilitates an interaction between three levels of reality, namely: the world (the city, the houses, the hospitals and the objects); photography; and the photojournalist’s intention. The photographs reflecting the emptiness of Alicante will be analyzed with regard to those three levels, although bearing in mind that, despite not being the complete reality, the photographs under examination do represent a plausible approximation to the existing reality and convey a clear ontological, ethical, political, aesthetic and sociological content.

**Figure 1.**
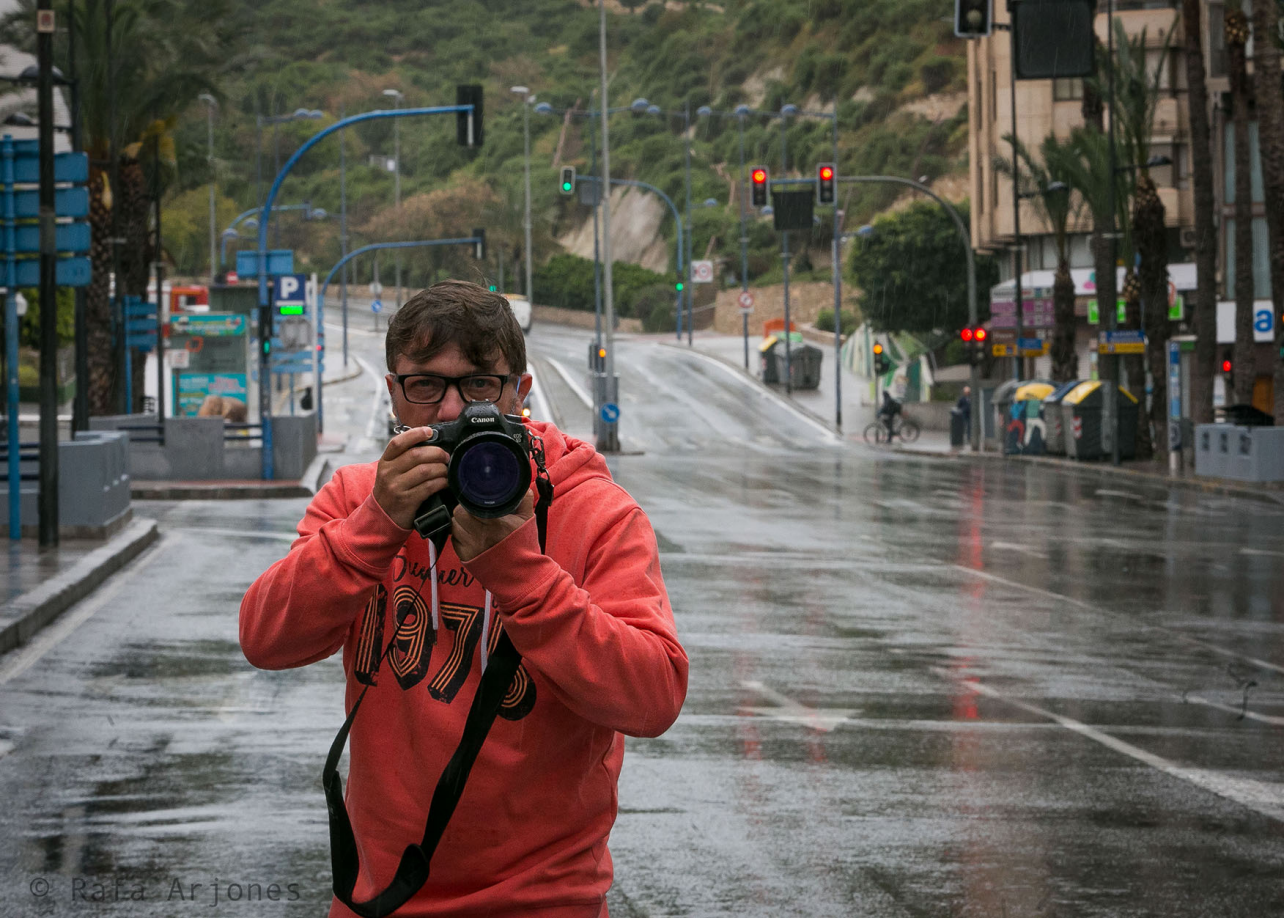
Rafa Arjones kicking the empty streets during the running of the bulls. This figure has been reproduced with kind permission of the author.

It should be highlighted that the 13 selected photographs were taken by the photojournalist Rafa Arjones ( Figure 1), who has been a professional photographer since 1987 and belongs to a family of photojournalists from Galicia that settled down in Alicante (with a population of 330,000 inhabitants and the capital of a dynamic province located in Southeast Spain). Arjones works for Información, Alicante’s most read newspaper and arguably one of the most outstanding within the Prensa Ibérica Group — the largest journalistic holding in Spain — and he also graduated with a degree in Photography and Visual Arts at Elche’s Miguel Hernández University (Alicante, Spain), in addition to which he studied audiovisual journalism, and was awarded with the Fotopres Prize by Fundaciò La Caixa in 1987. In order to select the photographs, we accessed Información’s newspaper and periodicals library, where we consulted and read all the newspapers published (66) in the period comprised between March 14th and May 18th, 2020 (corresponding to the first state of emergency). To be more precise, incorporating the keyword “Rafa Arjones” allowed us to obtain a total of 420 entries where his photographs were published. Nevertheless, those entries included many repeated images, because they had been published on several dates and/or in different regional versions of the periodicals (eg. Alicante, Elche, Alcoi, Vega Baja) to complement the corresponding articles. Furthermore, these images collected from Información were contrasted with those kept in the photojournalist’s own archive, which provided us with 102 images that, according to him, were the best and most representative of his professional career. He organized them by themes which revolved around the following concepts: “Empty city” (9), “Solidarity” (28), “Lockdown Surveillance” (7), “UME (Spanish Military Emergency Unit])” (5), “Rich Neighborhood-Poor Neighborhood” (11), “Reporters on the street” (6), “Disinfection” (5), “Healthcare Staff” (11), “Lockdown” (13), and “Queues in the supermarket” (7). Our final selection therefore includes 13 images (approximately 12.7 percent of the total) which, in our view, can be classified as representative and significant both in relation to the topics being studied and concerning Arjones’s way of thinking, as well as the events of the lockdown. Nonetheless, in order to achieve our aims, we paid attention not only to the general context of the pandemic — its main characteristics and effects — but also to the captions and to the written information offered by the journalist together with the image itself. Despite all our efforts, we are fully aware that the chosen corpus — as it usually happens — is undoubtedly small and, consequently, arbitrary. However, we strived to ensure that the selection included representative and meaningful images, or different expressions, and that they portrayed the most important issues and values with which Arjones observed Alicante’s urban reality and that, in parallel, they reflected in the most objective way possible the real circumstances in which the photographer worked during that period. Therefore, these 13 photographs lie at a crossroads between the photographer’s subjectivity and the objective goal of showing the historical reality of a city like Alicante during the lockdown. This article is accordingly framed within Weber’s Comprehensive or Interpretive Sociology, which regards the social world and the relationships generated by it as being meaningful. It also draws on applying the postulates of Visual Sociology to photojournalistic images taken during the lockdown. And last but not least, it takes into account that these journalistic photographs are social documents that grant what Weber refers to as “social schemes or types” and which, despite not exactly being the reality determined by coronavirus, allow us to better understand that specific reality.

## Results and discussion

### The emptied city in the context of the pandemic: A natural and social pandemic

COVID-19 has expanded globally and transnationally ^38^ because globalization, its networked structure or order and its multiple flows, along with urban and territorial organization, have favored its dramatic spread. In fact, people not only inhabit densely populated areas and live in multi-family buildings but also use public transport on a massive scale, all of which has largely increased the proximity of bodies. That is why the measures adopted by different states to “protect” their citizens against the risk of death have made 2020 a year that we are experiencing as an “historic” period with wide-ranging and unpredictable social, cultural, economic, environmental, ethical, and political consequences that especially affect individual and social bodies. No wonder this pandemic is a “disaster,”, a “catastrophe”, ^38^ a strange crisis which redefines uncertainty and risk, while disrupting daily life and its rhythms and directly reminding us of the congenital human fragility. ^39^ What is more, it amplifies this already unstable situation, insofar as nobody knows for sure where the pathogen came from. To complicate matters even further, this unprecedented and complex situation has not only stressed or collapsed public health systems but also interrupted or paralyzed economic activity all over the world. ^38^ Moreover, the merciless attack of the virus has forced nations to implement extraordinary measures pursuant to legislations, such as permitting the declaration of a “state of emergency”, which has curtailed basic human rights of citizens, amongst them: going shopping; demonstrating; attending events or participating in public activities; socializing; enjoying the open air; doing sport or meeting and hugging their loved ones. Alongside all of the above, the coronavirus crisis has placed science at the center of public space; ^40^ to such an extent that nations have adapted — at different speeds and with various levels of intensity — to different means that seemed useful to them in these circumstances: technical, scientific, and medical rationality, which even outplayed the hitherto dominant economic rationality, albeit only in the short term. At present, the prevalence is of measures such as social distancing, covering one’s mouth and nose, mass reclusion and care taken with hygiene. It might appear that, more than ever before, Society has become the space and time of rationality, order and security, whereas Nature would instead represent chaos, uncertainty and insecurity. However, it should also be borne in mind that the most recent coronavirus (SARS-CoV-2) is a product of the interaction between wild animals and human society. ^38^ Furthermore, it has both natural and social dimensions while simultaneously constituting a biological and social phenomenon ^41^ which is both chaotic and orderly, since it has generated pathogens — by Nature — that determine social norms and actions. That explains why 2020 is witnessing an intensified version of the old Western fight between Nature and Society, Culture and Civilization, ^42^ the only difference being that, for the first time in centuries, the latter three dimensions seem to be on the defensive and Nature is waging a fierce counterattack. While we wait for the final outcome, at the moment, Society has diminished in many respects and Nature, or at least a part of it, has grown rapidly.

### Spanish cities emptied and silenced

The current global pandemic has strongly affected Spain, one of the countries with the highest incidence of coronavirus as well as the highest associated mortality rates in the world: 27,709 deaths and 231,606 infected throughout the state of emergency in its home confinement phase (from March 14 to May 4, 2020). Although a variety of reasons may account for these particular conditions, Spain’s social and cultural context, built around interpersonal relationships where sociability, proximity, physical affection, and intergenerational family structures prevail, arises as a key factor. Bearing that in mind, the Spanish government’s decision to implement social distancing through home confinement as the main strategy to protect its citizens comes as no surprise. ^38^ This has had manifold consequences. Above all, the country has been silenced and the streets, squares, gardens, and stadiums, as well as the cultural and entertainment venues of the cities — including Alicante — are suddenly empty and completely deprived of any activity: no walkers, no music, no cars, no cinemas, no shopping centers, no beaches, and the usual social activities have been cancelled or modified in line with new government guidelines. In effect, social, cultural, and spiritual interrelations have deteriorated due to the social distancing of bodies, the confinement inside homes, and the cancellation not only of every kind of public cultural and sporting event — except for TV remakes and the artistic spontaneous activities meant to show solidarity on social media and on private balconies — but also of religious and political ceremonies. In turn, personal homes and hospitals have transmuted into a “Home-World”. ^43^ This is the result of a process in which the social universe has been placed within its four walls. Expressed another way, social, family and other relationships have been overlapped with the public activities of the so-called “home office”. In addition, family life has been reshaped through the organization of online virtual gatherings and the transformation of the living room or the bedroom into a virtual classroom. ^44^ Furthermore, the healthcare staff in hospitals not only had to care for the basic needs of the isolated patients but also helped them emotionally. The population has additionally been forced to share their day-to-day life together more intensely, even if it is through the Internet, arranging both family and social life around social media such as Instagram, Facebook or TikTok and popular messaging or communication-oriented applications such as Whatsapp, Zoom or Google Meet. ^45, 46^ This digitization of society has made social interrelations become more virtual and therefore less carnal than ever before. Moreover, certain emotions strongly arise in this context which, albeit lived in “isolation,” are shared in the households, from balcony to balcony, from call to call, from video to video, and from whatsapp to whatsapp. More specifically, what we all communicate in these spaces of shelter — homes, hospitals, and social networks — is ambivalent. On the one hand, people lay more emphasis on sharing their day-to-day routine as well as on the implicitly associated emotions, e.g. love, play, smiles, happiness or solidarity, the last of which has visibly materialized through the active mobilization of civil society to help those who needed it most in this crisis situation as well as through the intergenerational volunteerism initiatives undertaken in many towns and cities. ^45^ Likewise, expressions of gratitude and affection are being addressed to the new heroes of society, those everyday beings without whom chaos and tragedy would have taken over, namely: doctors, nurses, the police, the military, undertakers, drivers, transporters, pilots, supermarket cashiers, pharmacists and pharmacy assistants, and journalists. Nevertheless, alongside positive emotions, other negative ones have also developed such as fear, anguish, rancor, conflicts, gender and childhood violence — at home or on social media — combined with expressions of fear and racism, the unstoppable growth of Fake News, the revival of latent feelings of hatred and resentment, the exacerbation of political differences and the consequent social polarization.

## The images that reflect the emptying of Alicante and the emergence of the “Home-World” and the “Hospital-World”

### The empty city

**Figure 2.**
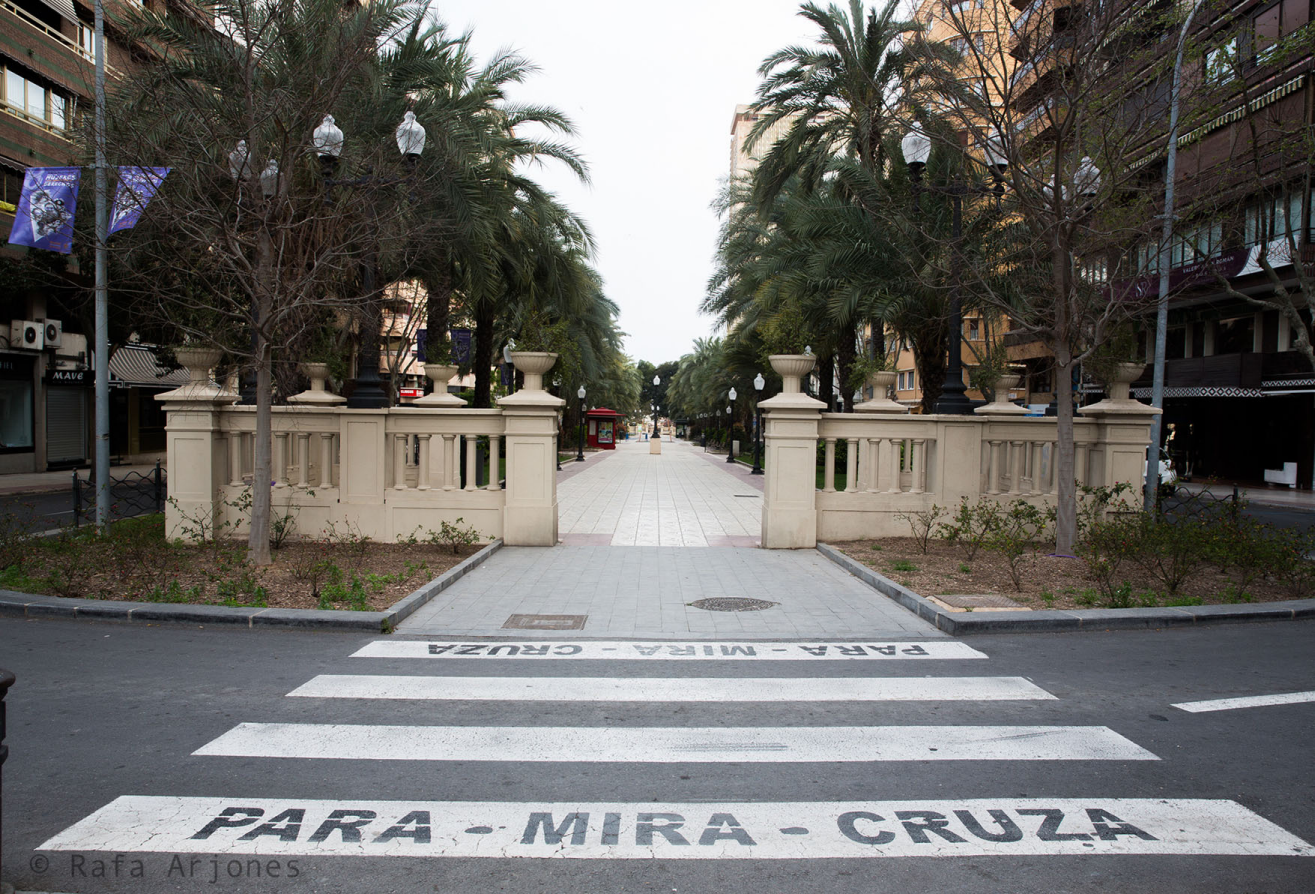
The image shows one of the busiest streets of the city of Alicante emptied. People remain, therefore, in their homes. This figure has been reproduced with kind permission of the author.

Figure 2 shows a tree-lined walk of a central street in Alicante with a perspective towards the commercial area of Maisonnave Avenue, where you can find El Corte Inglés (department store) and numerous stores in which products such as clothes, shoes, perfumes or jewellery, amongst others, are sold. The photograph is taken in the middle of the day, without any passers-by and with no cars moving around. The photographer neatly captures the symmetry of the whole area, with two lampposts and two trees visible on both sides and, in the center of a stone balustrade, there are two sorts of jambs resembling a door which open in parallel to the walk. On one of the lampposts, some advertising banners are being blown by the wind. In front of them, exactly in the center of this geometric composition, there is a zebra crossing with parallel horizontal white stripes which, despite breaking the verticality of the other urban objects, gives continuity to the linear axis of the walk at the same time. The words “PARA-MIRA-CRUZA” (STOP-LOOK-CROSS) can easily be seen written on two of those stripes written in capital letters. The advertising has become useless, since there is no one around who can see those banners, as has the commercial street, insofar as its businesses are closed and consumers have disappeared too. The economy finds itself at a standstill, the same as the urban dynamics, activity, and social interrelations. In short, this space has undergone a minimization process, ceasing to be understood as a culturally affective and symbolic space, ^47^ and time has come to a standstill around it. On the other hand, the vertical and horizontal coordinates along which social life flows, as well as the norms dictating it, have suddenly become outdated, as they do not seem to be valid for the current circumstances brought about by the pandemic: the absence of individuals who could comply with them turns those rules into ineffective regulations whose existence is absurd.

**Figure 3.**
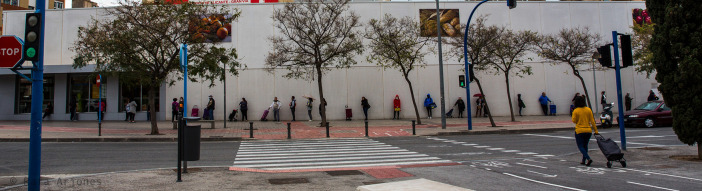
Long horizontal facade of a supermarket. People are seen waiting to be able to buy and keeping the presribed safe distance. In those moments, when it was possible to go out to the street, waiting becomes one of the main moments in which there is a certain social relationship. This figure has been reproduced with kind permission of the author.

In front of the horizontal facade of a supermarket, there is a long line of people waiting to enter and do their shopping whilst complying with the social distancing rules ( Figure 3). Most of them have a shopping trolley, two of them an umbrella, and another one a hooded anorak; some are looking at the person standing in front of them, whereas others gaze at the camera lens. An empty zebra crossing stands out in the center of the photograph, and across the street, there is a stop sign and a green light to signal the non-existent cars. Leafless trees arranged along the same sidewalk occupied by the future buyers, all of whom are standing, and lower iron pylons around the zebra crossing, together with lampposts and traffic signs, all of them in a vertical position, complete the main scene. Finally, on the right of the zebra crossing, and seemingly unwilling to cross it, a woman with a shopping trolley with her back to the viewers takes a shortcut towards the line. The citizens standing still on the wall are organized following a calculated and orderly arrangement which is coherently reinforced by the vertical and horizontal lines of all the other objects in the photograph. Thus, the usually chaotic movement of city dwellers has been replaced by a group of paralyzed individuals whose apparent lifelessness makes them look like any other inert object. In fact, these people now form a motionless part of the townscape — exactly like the lampposts, the pylons, and the leafless trees — as if coronavirus had absorbed their social, and also to a certain extent their individual body energy, which only manifests itself in the different stances or ways of waiting. Attention should likewise be paid to the touch of chaos and individual “indiscipline” transmitted by the walking woman who has chosen not to go across the zebra crossing in the normal way.

**Figure 4.**
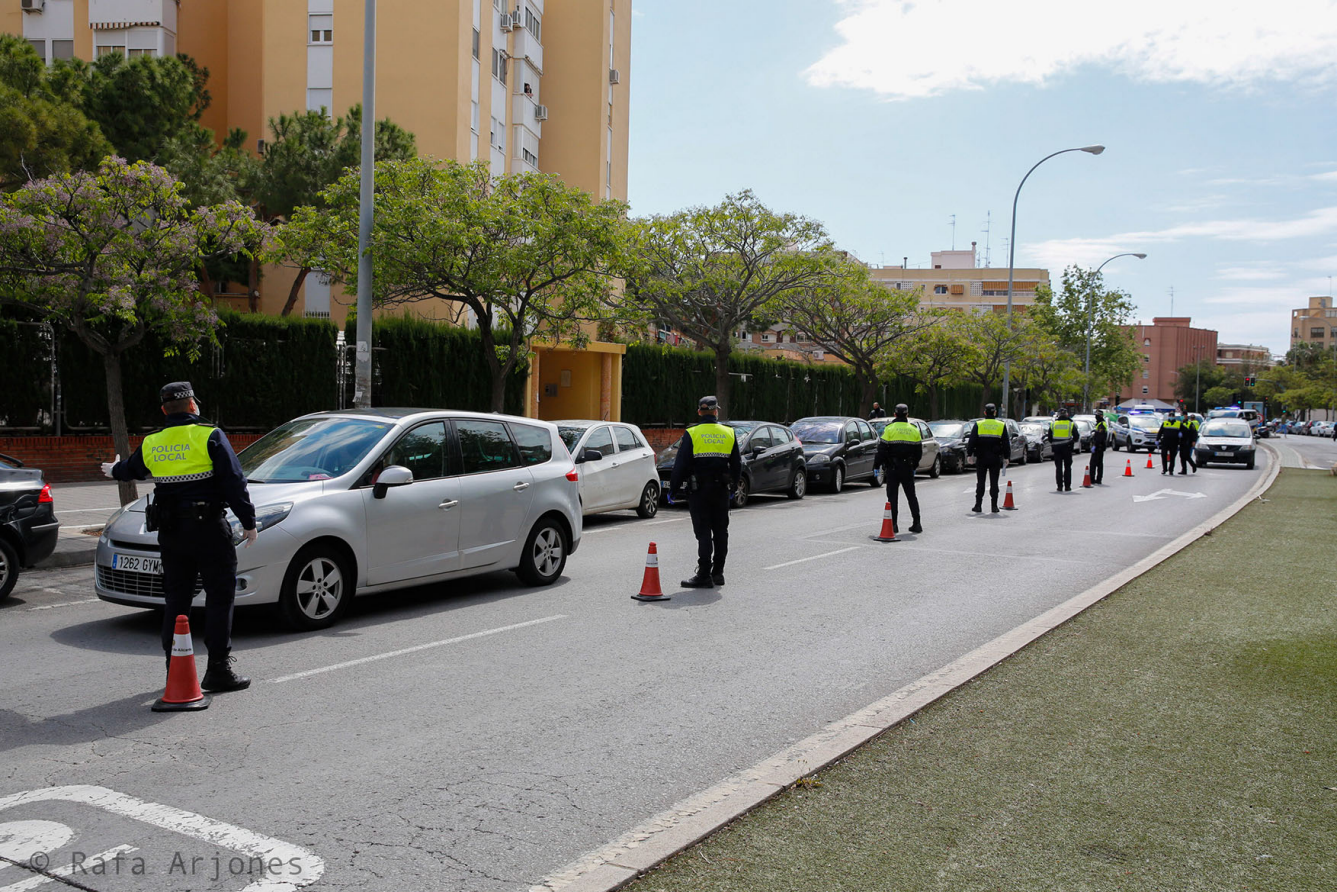
Eight municipal police officers inform the population of the control measures established by the health authorities. This figure has been reproduced with kind permission of the author.

The eight municipal policemen in Figure 4 are also arranged in order, and separated pursuant to social distancing rules, in the middle of the day. What is more, they try to ensure that confinement regulations are being abided by and, more precisely, that the cars on the road have the corresponding authorization to circulate. The first of these policemen on the left of the photograph, has stopped a vehicle, probably to check whether the driver has such an authorization or not. As the case may be, the driver will be allowed to continue, warned that the quarantine is not being complied with or even fined for the possible infringement committed. Both the diagonal line on the road and the one formed by the standing officers seem to head towards a city street that the vehicles are coming from; hence why all the policemen are looking in that direction instead of paying attention to what is happening on the opposite side of the same street. As attested by this photograph, COVID-19’s virulence has made it necessary to apply social surveillance and control measures, since what is really at stake is survival through the maintenance of social institutions, especially economic, health and democratic ones. The problem with this virus precisely lies in the fact that it attacks not only the regulated social organization and its dynamics, but also the density of contacts, affections, and interrelations, as well as our individual biology. To counter this, and following scientists’ advice, the government has imposed discipline on physical bodies to prevent them from coming into contact and to encourage society as a whole to apply self-control and energy-restriction measures, ultimately seeking to prevent the virus from being fed and accordingly allowed to spread freely. Two questions may be posed in this regard, though: (a) is the problem being addressed head-on (the policemen are looking in a single direction)? and (b) has society not been reduced to a certain extent as a result of this?

**Figure 5.**
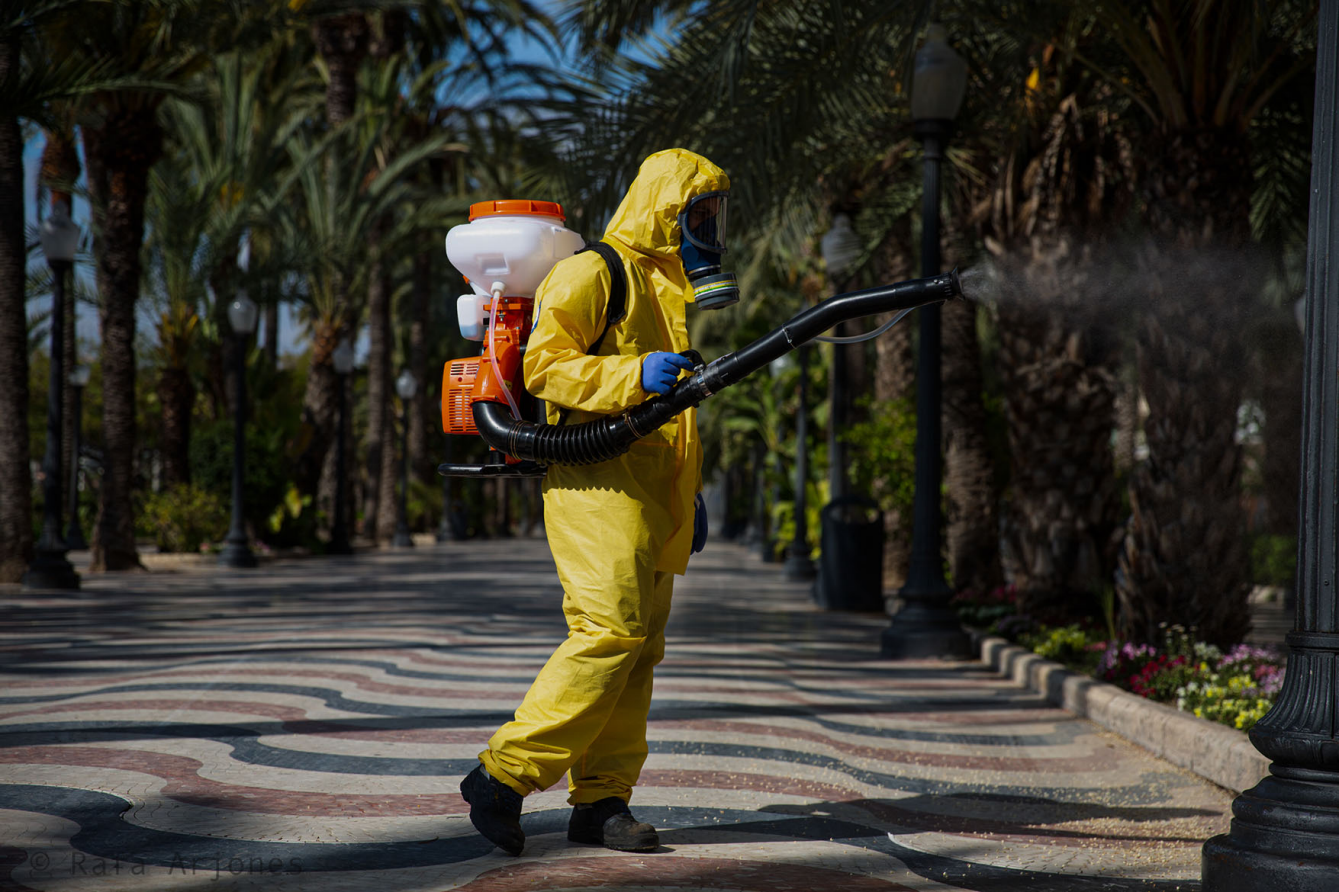
A worker cleans the streets with the aim of reducing the possibility of infection and also to reduce environmental virus particles. The lack of knowledge about SARS-CoV-2 led the authorities to implement various measures without knowing their real effectiveness. Subsequent rapid advances in knowledge led to social confusion about these decisions. This figure has been reproduced with kind permission of the author.

**Figure 6.**
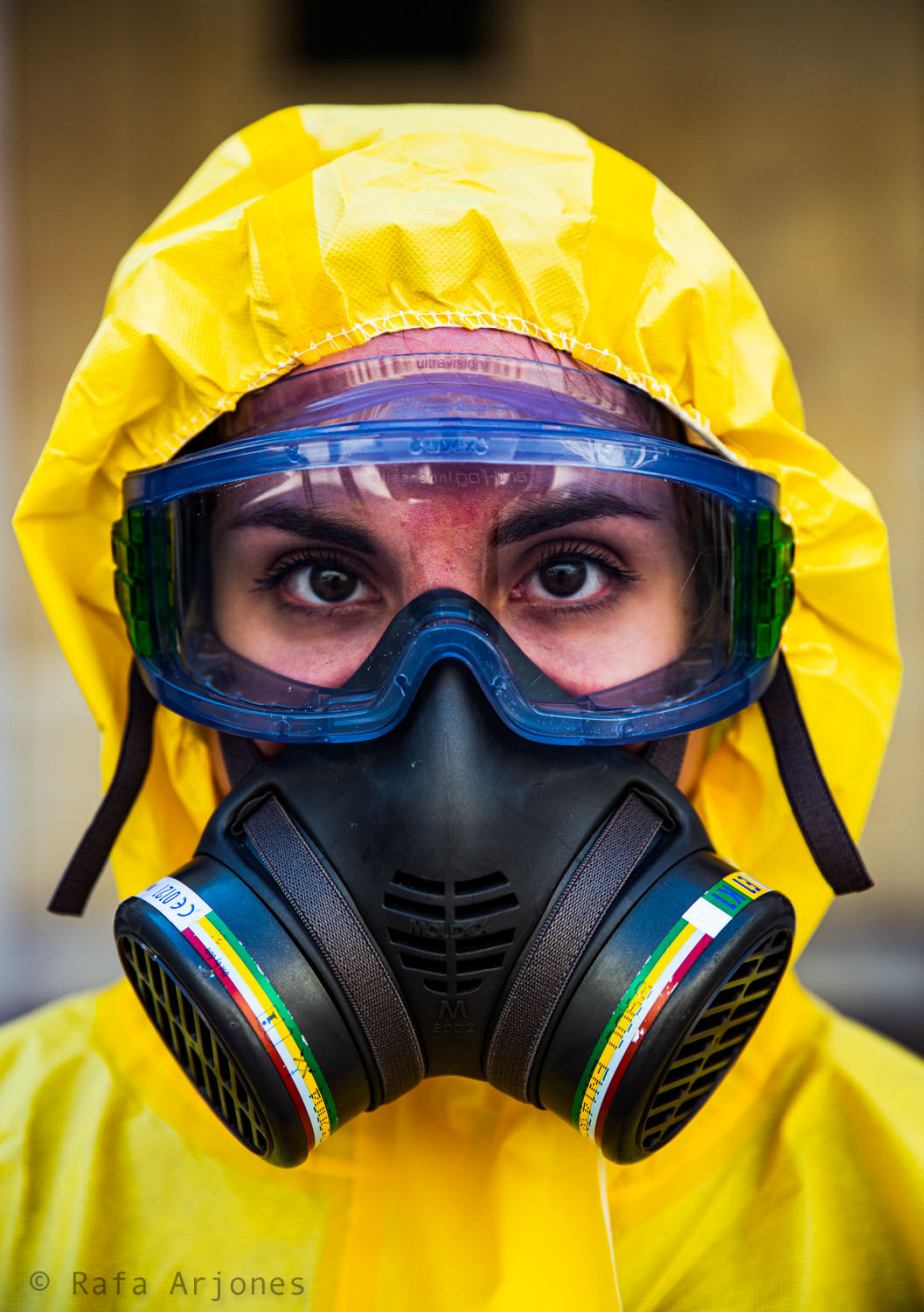
A member of a cleaning team wearing Personal Protection Equipment (PPE). This figure has been reproduced with kind permission of the author.

**Figure 7.**
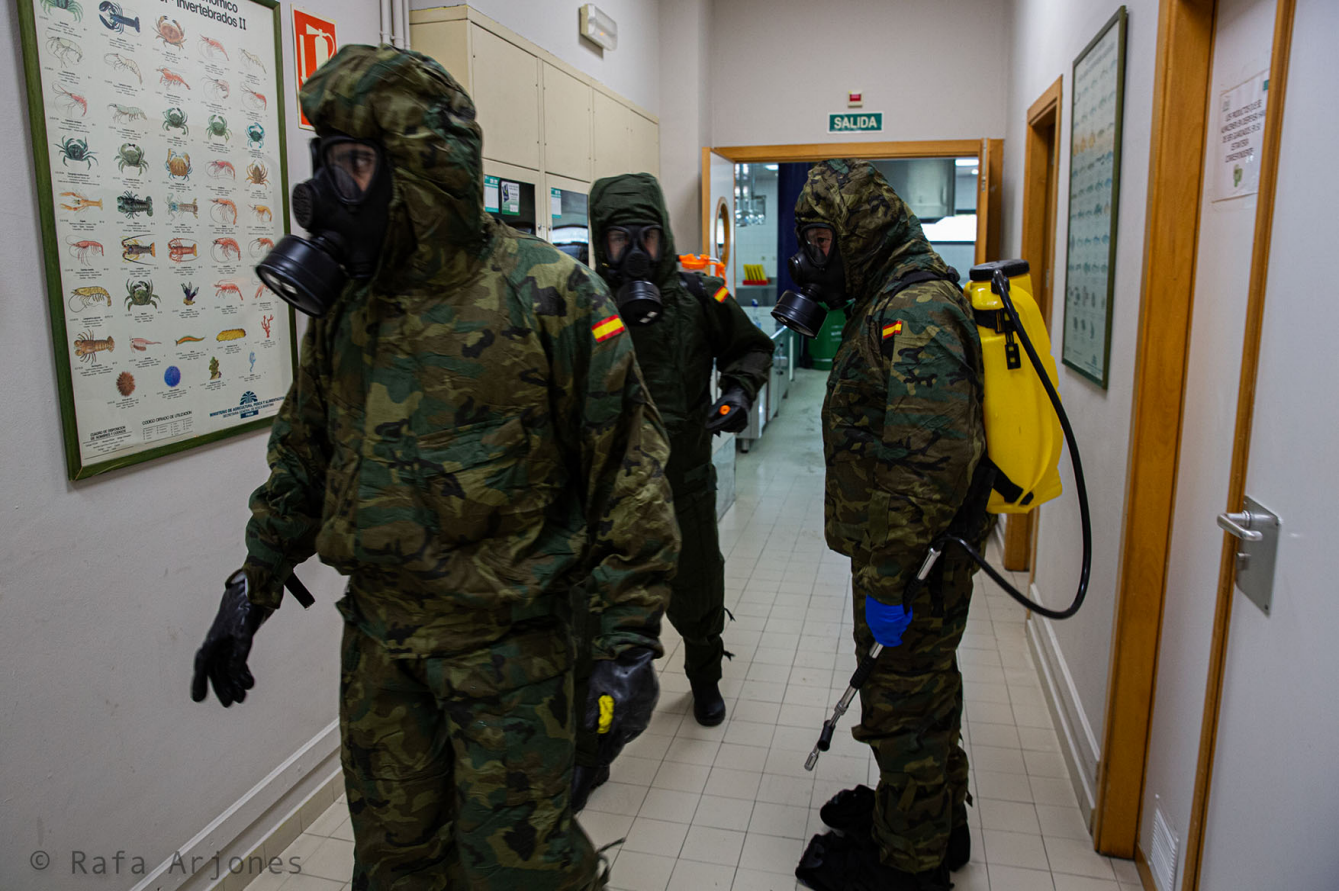
Three soldiers from the Military Emergency Unit wearing protective suits that make it difficult to identify their corporeality. What it can be seen are uniforms that indicate their status and social role. The involvement of the military in the protection processes generated a certain identification of the military with the heroes. This figure has been reproduced with kind permission of the author.

The popular *Explanada*, Esplanade, in Alicante, usually full of people, wandering passers-by, families, children, seniors and tourists, is now empty ( Figure 5) despite the fact that it is daytime. We can only see, right in the center of the composition, a private company worker wearing a yellow protective suit, a mask, and gloves who is fumigating. Although he is standing, his position is perpendicular to the orientation of the walkway, marked by the linear and vertical arrangement of the palm trees, with which he also contrasts due to the horizontality of the device in his hand. The geometric coordinates that represent order in society are once again subverted by the situation, which in turn alters the function normally associated with a walkway like this one. Why fumigate if nobody is strolling in the area and there will be no one for weeks to come either? And, more importantly, will the amount of chemical dumped onto the street be effective and sufficient to destroy the virus in such an open space? Do these actions not appear to reflect the state of confusion in which this situation has immersed us, our scarce knowledge about the pandemic or about what really needs to be done in order to fight it? On the other hand, this worker’s loneliness becomes evident, since he is now the only inhabitant of this city, an anonymous tragic hero — we cannot see his face — who risks his life to keep the city clean, without knowing for sure if his effort will eventually turn out to be useful or useless. Figure 6 also shows the individuality of the young woman portrayed in the foreground, dressed in a yellow protective suit that covers her hair and wearing an anti-pollution mask as well as protective glasses. Behind them, we can see her large dark eyes wide open and looking directly into the camera, seriously scrutinizing or expressing her state of mind and/or the gravity of the situation. The skin allergy on her face, above her nose, most probably caused by (wearing) the mask, might also be a psychosomatic effect of the stress she is experiencing. It should also be highlighted that her mask resembles those used by soliders in war to protect themselves against polluting gases, the only difference being that our enemies are not other human beings now; we are waging a hand-to-hand combat against nature, embodied in the virus. In any case, its harmful effects can hardly be denied, insofar as COVID-19 affects not only the health of bodies but also that of minds. The next photograph ( Figure 7) shows three UME (Military Emergency Unit) soldiers wearing their military outfits, masks that cover their faces completely, and gloves. In this case, they are disinfecting an educational center, but it might as well have been a private residence (it should be remembered that in Spain there are a number of old people’s homes managed by the State and the regional governments) or an airport. Rather than individuals — defined by faces with unique features — and human bodies, what we see is uniforms indicative of their social condition, social roles, and anonymous heroes who are working for the benefit of public health, putting their own lives at risk.

### The “Home-World”, the “Hospital-World”: individualism and communitas

**Figure 8.**
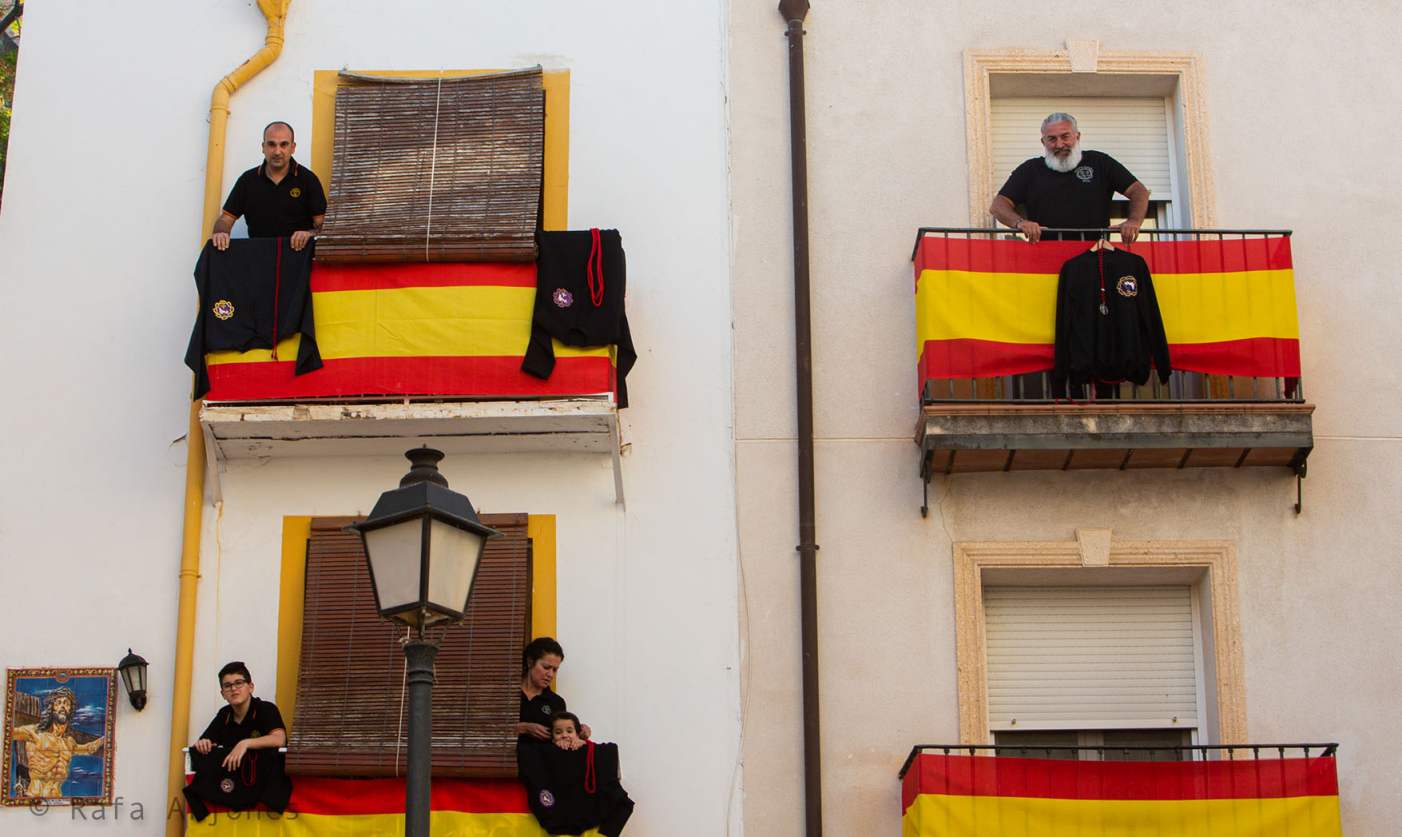
Facades of two buildings with four balconies adorned with the Spanish flag. The pandemic also increased certain patriotic spirit. After all, it was a symbolic war against the virus. This figure has been reproduced with kind permission of the author.

Figure 8 shows the facades of two buildings in the Santa Cruz neighborhood — a typical area of Alicante’s historical center — and, more specifically, four balconies adorned with the Spanish flag, three of the balconies are adorned with the black outfits of the Holy Week brotherhood and are occupied by five people — three men, one woman, and a little girl — dressed in the same color and outfit. All these inhabitants of the neighborhood whose black color stands out from the white or light-colored walls seem serious, including the young girl, and appear indifferent to one another, even those who share the same balcony and must be relatives. The mother, on the other hand, makes a gesture to approach her daughter, who barely peeks through the railing and over the ritual garment while attentively looking at the street. No one occupies the fourth balcony, and there is no hanging black outfit either, although the window ajar suggests that its owners might be inside. Finally, a tile with the body of Christ can be seen on the white front of the left hand-side building. The presence of Christ, added to the scene with the dark ritual costumes on the balconies along with the ritual and festive clothing of the neighborhood inhabitants, constitutes a physical embodiment of the festivity both in the actual buildings and in those who inhabit them. Truth be said, though, the sad faces express the emotional frustration for the inability to parade, with fervour and full of an “effervescent”, “emotional” and “integrating” energy, the *paso* (scene/image) of the Reclining Christ through the streets of Santa Cruz. In fact, the people in the housing blocks do not seem to care about one another, all of them absorbed in their own disillusionment or bewilderment, and indifferent to whatever their next door neighbours may be doing and experiencing emotionally. Meanwhile, the neighbourhood is empty; nobody stands outside the houses, there are neither women nor men, children, elderly people nor tourists, which means that the festivity has been left hanging from the balconies due to the impossibility to descend and parade down the street.

**Figure 9.**
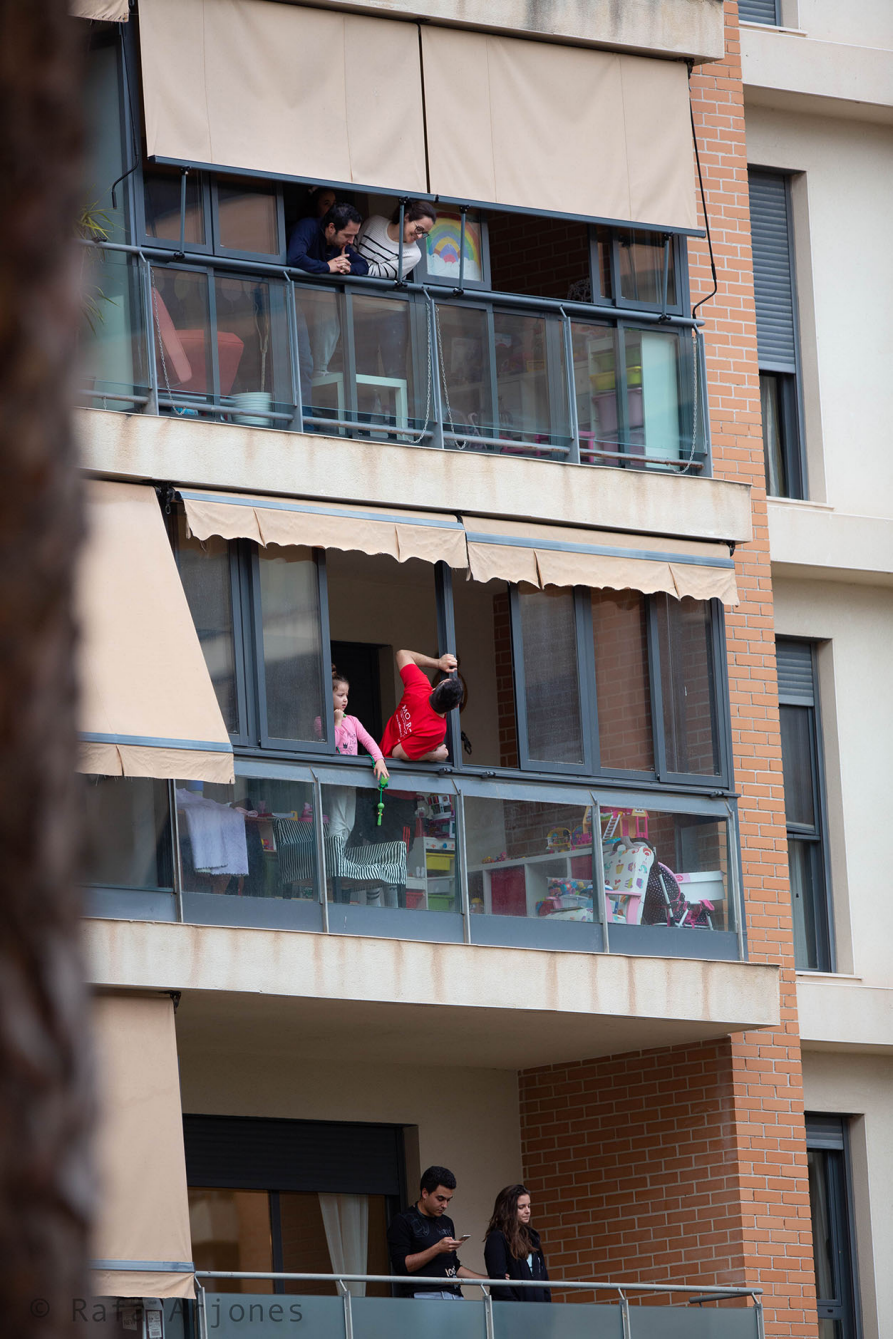
Home confinement increased social relations between neighbours and generated different ways of shaping the *communitas.* The image shows how people talked from their balconies when they were unable to go out into the street. This figure has been reproduced with kind permission of the author.

The interpersonal relationships in the building in Figure 9 are very different. Some people occupy the balcony on three of the floors. On the lower balcony, we can see a young couple somewhat separated from each other, whose bodies do not touch; he is looking at his mobile phone while she observes the street. The balcony on the flat above them shows a girl and a man whose distorted body is turned upwards while he leans on the window frame. He is chatting happily with the couple on the top floor, as suggested by the smile of the woman who directs her gaze downwards. A poster with a rainbow drawn on it is glued to the windowpane by her side. These social interrelations which seem temporary and somewhat forced, coexist with those of indifference or self-absorption represented by the couple on the bottom floor. Furthermore, the rectangular space of each terrace, together with their glazing and awnings, separate the respective flats which despite being arranged one on top of the other, are autonomous. This is reinforced by the fact that they are not at street level, but above it, which keeps residents far from the normal daily urban pulse. Furthermore, the space where these flats are located represents the edge of the construction, the limit between the inside and the outside, between the society of individualization and the family, between intimacy and the expression both of emotions and of social interrelations with other people, and between the household and the street. For this reason, every day at 8:00 p.m, they ritually applaud the professionals who are risking their lives, greet other neighbours, show their attachment to traditions, participate in some cultural activities or call on the population — as denoted by the rainbow which has become synonymous with the healthcare profession during this crisis — to become aware of the need to stay at home for the sake of collective health and to be hopeful that they will successfully overcome the critical situation in which they find themselves.

**Figure 10.**
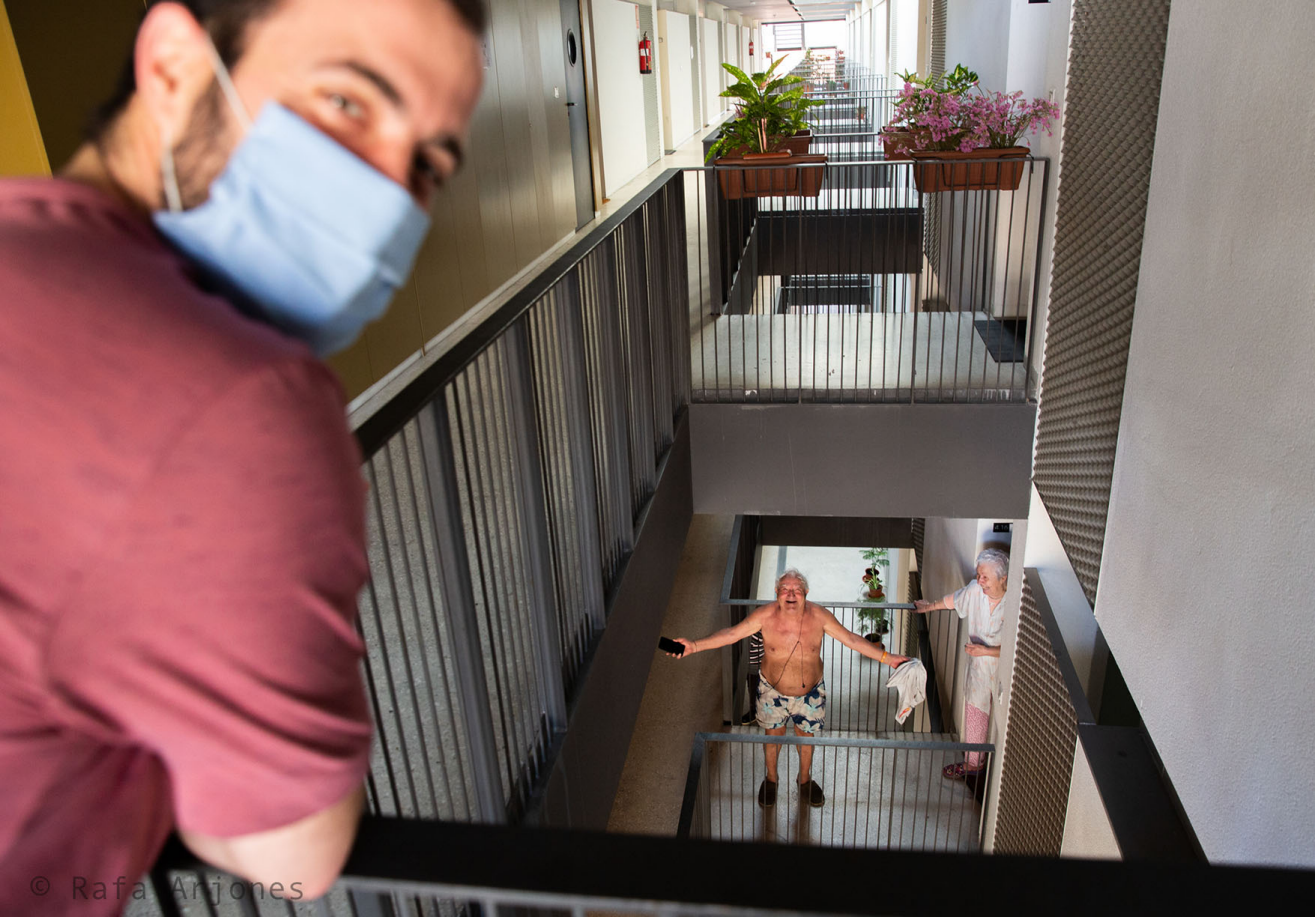
Another strategy used to increase social relationships while reducing the likelihood of contagion was to communicate via the stairs of a building. People seek to promote social interaction as a reaction to a mechanism that was intended to promote a degree of temporary social isolation. This figure has been reproduced with kind permission of the author.

On the landing inside a block of apartments in Figure 10, a young man wearing a face mask and a short-sleeved shirt is seen in the foreground as he turns his head to look at the photographer and tell him what is happening there. On the same floor, a very long horizontal corridor opens in which the flat doors are separated by openings and handrails and where someone else can be seen, blurred and distant, in the background. Downstairs, there are two elderly people, one in underwear or swimming trunks and shirtless — he carries his shirt in one hand and his cell phone in the other — and a woman leaning against the door of her home wearing a housecoat. Both look at the camera too, and the older man seems to show, amusingly opening his hands, how he is dressed, or undressed. The segmentation between neighbours in the design of this building follows a horizontal pattern organized around the openings and metallic handrails, in such a way that the flats are separated not only by their inner walls but also by the architectural elements of the corridor, without forgetting the invisible virus that also forces them to stay apart. In the scene, the main three protagonists seem to establish a communication between accomplices: the young man prefers to look at the photographer and, the same as the older man, speaks to him, while his wife observes the photographer with an expression situated between amusement and amazement. Finally, the person in the background is so far away that he remains totally unaware of what is going on a few meters away. On the other hand, the neighbours are outside their houses, in the corridor, albeit dressed as if they were inside their homes or on the beach, which attests to the confusion between what is inside and outside, what is intimacy and “extimacy” and, in short, what it means to be imprisoned — reflected in the numerous bars of the banister — to be safe or to be free to run risks.

**Figure 11.**
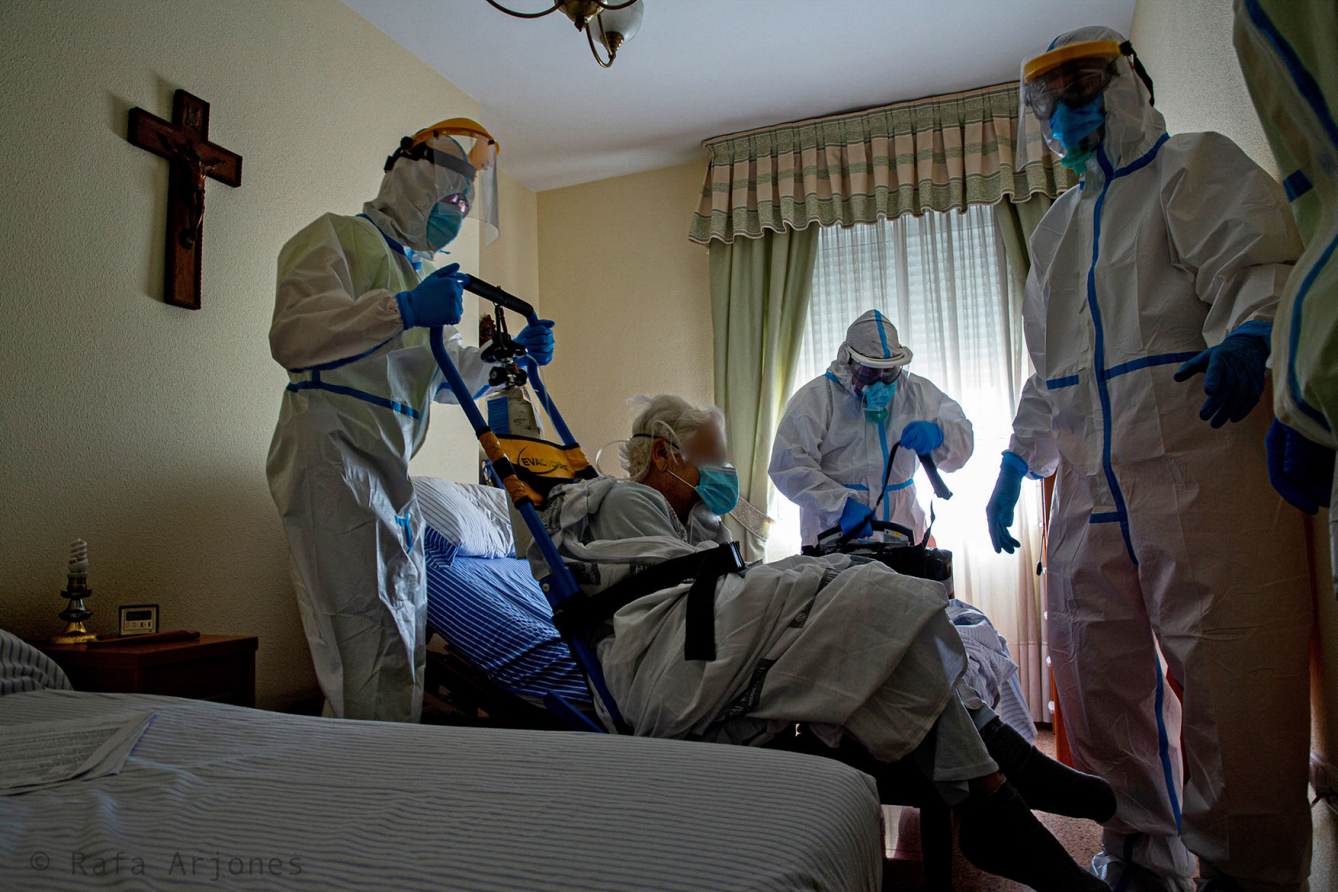
At that time, health measures were greatly increased. In the picture you can see how a small room was occupied by several people in order to clean and keep the sanitary situation under control. In the process, the social space becomes (symbolically) an alternative “health centre”. This figure has been reproduced with kind permission of the author.

**Figure 12.**
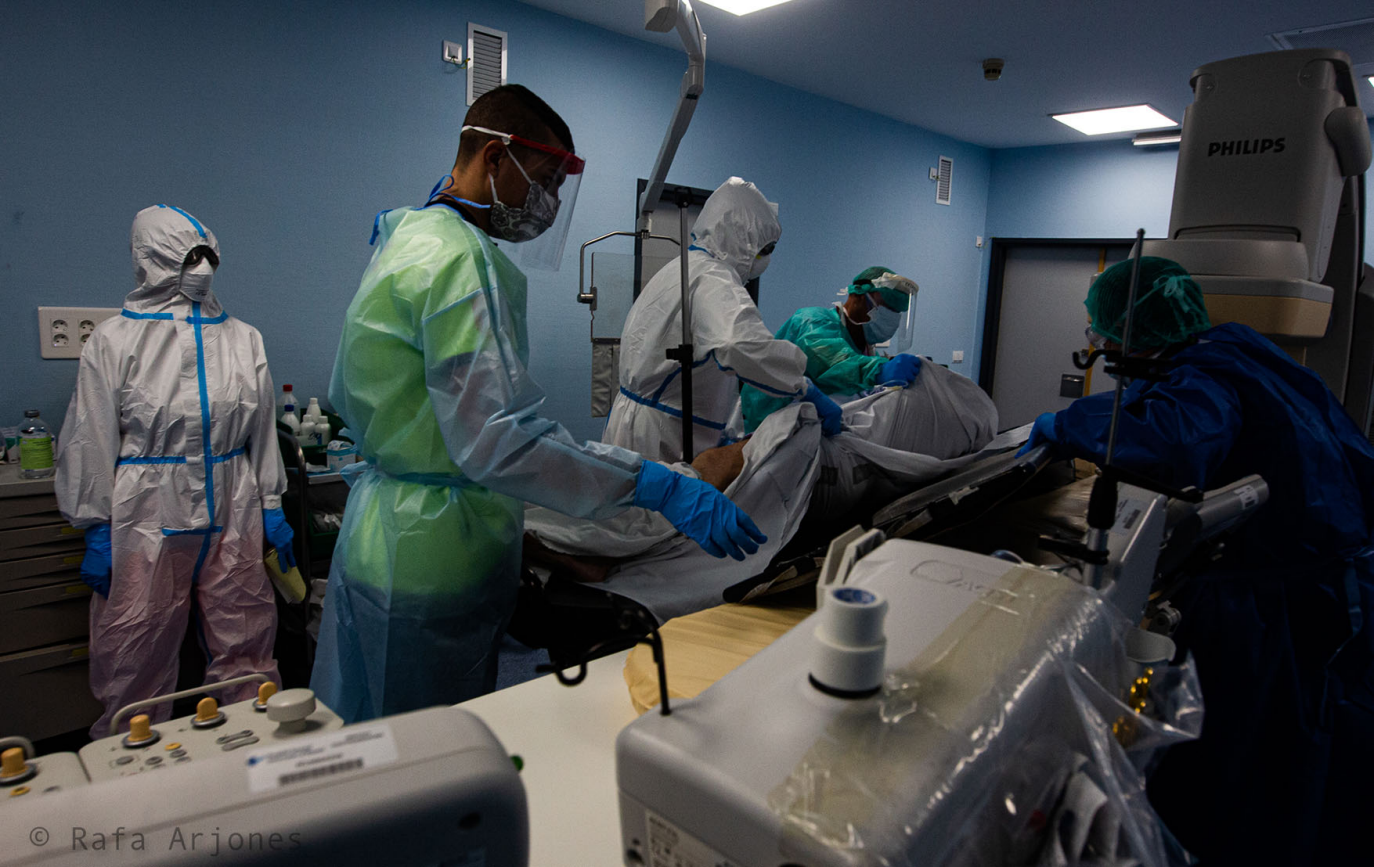
The pressure on the social system is increasing. Lack of knowledge about the virus leads to an increase in health measures. This is in addition to the numerous cases of people affected by respiratory problems. In this image, hyper-protective behavior of the healthcare professionals clearly suggests that the patient is infected with COVID-19. This figure has been reproduced with kind permission of the author.

**Figure 13.**
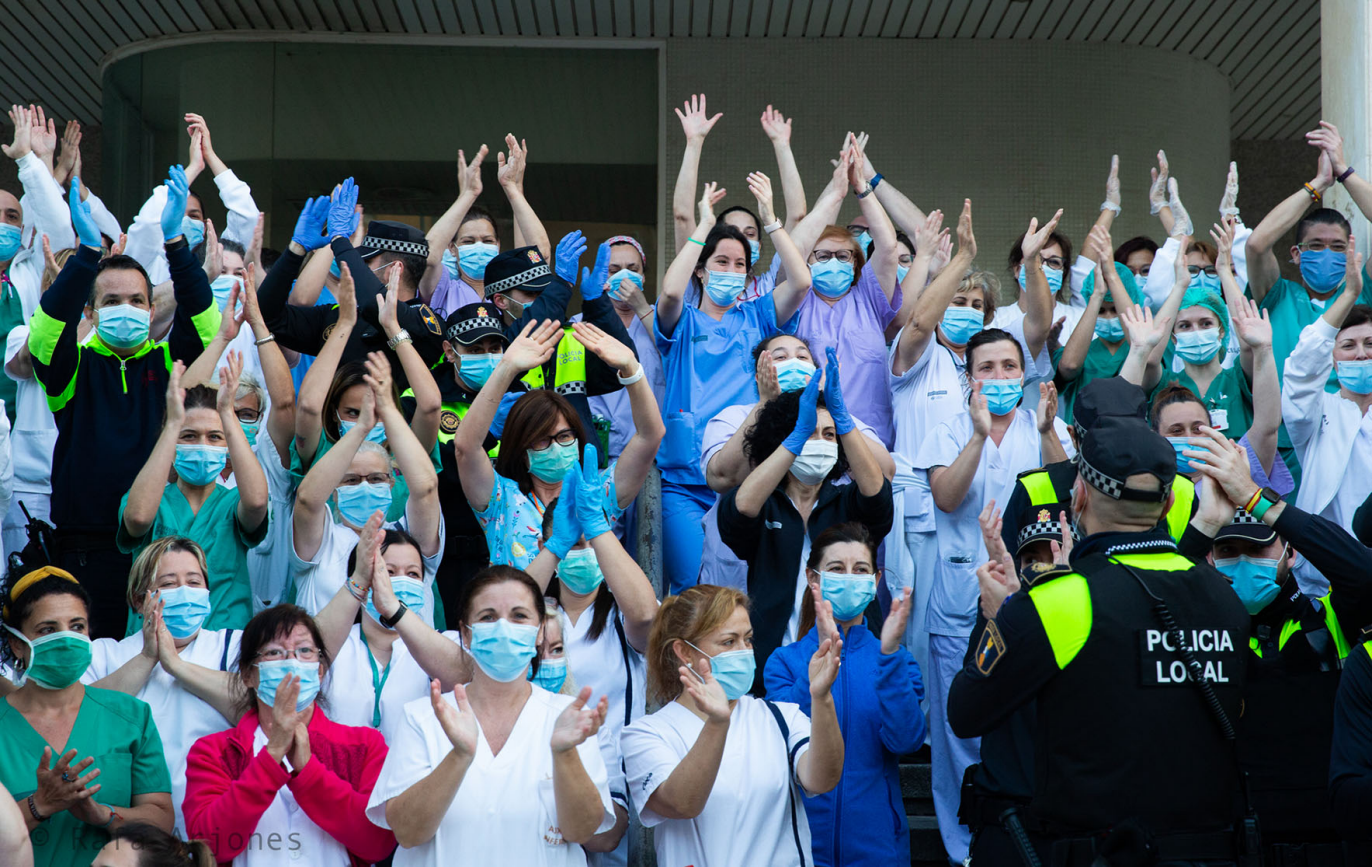
During the confinement, a social phenomenon became widespread, which was intended to show the appreciation of the population for the hospital workers: clapping. In response to this phenomenon, hospital workers would go out to the hospital gates to clap and express their gratitude to the population for its social behaviour. This figure has been reproduced with kind permission of the author.

A narrow room ( Figure 11) in an retirement home on whose white wall we can find a large crucifix and a window with its closed white curtains that prevent us from seeing the outside, although a sifted light manages to come in. This relatively dim room is almost entirely occupied, with hardly any space left, by three healthcare professionals wearing white protective suits, a mask and gloves, who are about to transfer an elderly woman to the hospital, who also wears a mask and sits on a folding chair. Is she infected with coronavirus? In such a small and unventilated space, it is easy for the virus to flood the room and infect its occupants, without the dark cross being able to do anything about it, other than announce the proximity of a possible future death. In nursing homes it is usual for one or two people to be responsible for cleaning the residents’ rooms. In contrast, the photo shows three people cleaning the room. This generates a symbolic process of transformation of social environments into biomedical ones. It also shows the commitment of public institutions to public health. In fact, during the strict confinement, the authorities sent external staff to the nursing homes in order to protect the most vulnerable. Figure 12 shows a hospital room with equipment and six people, five of whom are healthcare workers wearing suits, masks and gloves, and the other, someone lying on a stretcher almost completely covered by a sheet, so that only one leg can be seen. The hyper-protective behaviour of the healthcare professionals clearly suggests that the patient is infected with COVID-19, whereas their different gestures and bodies denote action and concentration. On the left side of the picture a standing healthcare worker can be seen. Despite apparently observing the scene calmly and motionless, her leg is advancing in a way which reveals that she may approach the patient or move towards any of the machines or objects in the room immediately. Time to act is compressed, minute by minute, although chaos, urgency, accelerated body movements and the flow from life to death unfold in a context of heightened uncertainty. In Figure 13 we can see a motley group of nurses, doctors, cleaners and service staff, as well as local police in their uniforms, mixed amongst one another, at the door of a hospital. Most of them are raising their hands to draw attention to the dramatic situation that they are living through or applauding as they look to the camera, with the exception of some policemen with their backs on the spectators who are looking at the healthcare staff and applaud them by way of a heartfelt tribute. The photography transmits great dynamism and, despite the absence of joy in the group, there is a strong emotional energy as well as a powerful conviction that they are doing the right thing and, more importantly, a will to do whatever it takes both ethically and based on solidarity to help anyone who may need it. Moreover, by standing so close, without any social distancing, they demonstrate the inseparable unity and huge strength of this group and, in short, make it clear that the only possible way to cope with all this is working side by side, joining hands, bodies and brains.

## Conclusion

In our view, the analysis of the photographs taken by the photojournalist Rafa Arjones has allowed us to confirm the three key consequences of the COVID-19 lockdown in Alicante. The first one, that the city was deserted, its streets uninhabited, its stores and promenades closed, without any vehicles or pedestrians ( Figure 2). The people who could leave their homes did so only to buy the essentials to eat ( Figure 3); the lonely ones took to the streets to work e.g police ( Figure 4), fumigators ( Figure 5 and Figure 6), the military ( Figure 7), healthcare staff ( Figures 11, 12 and 13), drivers, pharmacy and shop assistants, sweepers all became heroes who risked their lives to decontaminate, to attend to the sick, to ensure safety or to maintain the essential services. The Alicante-born photojournalist’s carefully taken images likewise emphasize a neat geometry framed within two coordinates — horizontal and vertical — and a central axis of the composition around which the portrayed objects and persons are distributed ( Figures 1, 2, 4, 8 and 9). He does so because one of his discourses refers to how the critical situation triggered by the pandemic has disrupted the social order and the norms guaranteeing it which that geometry represents. It is as if questioning the coordinates and axis that sustain current life social space had resulted in a collapse of society itself. In fact, its content has been radically altered, since social interrelations, the economic dynamics, urban vitality, affections and the contact between bodies, together with the energy which renews the citizens’ pulse, have been minimized, thus emptying society. At the same time, control and surveillance have increased with the aim of keeping us safe ( Figure 3). Therefore, representative democracy has somehow entered “quarantine” ^48^ and has been partially emptied as well.

The streets have been emptied while homes and hospitals — society’s last shelter — have been filled, expanding their traditional functions to become a more or less safe “world” where both the usual activities and new ones can take place. Thus, the rituals ( Figure 7), the manifestations of solidarity, and the conversations that used to form part of street life now develop in balconies or corridors ( Figures 7, 8 and 9), narrow, enclosed, and segmented areas where the new forms of loneliness, self-absorption, estrangement and lack of affection also become visible. Hospitals ( Figures 10 and 11) have in turn assumed new rituals of empathy, providing social cement and loving care for the sick, and their staff have acted as a true family for patients during these times of loneliness and forced isolation. The photojournalist has successfully captured human emotions typical of such a critical situation in homes or hospitals, amongst them the disappointment due to the impossibility to enjoy parties and rituals ( Figure 7), the seriousness ( Figures 5, 7, 8 and 12), the estrangement and uncertainty associated with this situation, the tension, the concentration ( Figures 10 and 11), the empathy, the solidarity, and the firm will to protect loved ones and to look after the most vulnerable people. As for the street, it has lost almost all of its traditional functions, while the balcony ( Figures 7 and 8), the corridor ( Figure 9) or the hospital door ( Figure 12) have assumed its competences to a large extent. For this reason, they have been transformed into a space of communication or isolation, of ritual, of solidarity, and of externalization or containment of emotions. They have become a new liminal space used in the three successive phases — separation, marginality, and aggregation ^49^ — or moments of transit that characterize the rites of passage of ancient societies. ^50^ They now constitute the door jambs through which citizens confusingly go from the inside to the outside, from the public to the private, from the normality of the past to the uncertainty of the future, from vitality to the death of the social, perhaps so that — the same as in rites of passage — a regeneration takes place and a new stage can begin.

The confinement of the population has been accompanied by an increased individualism and, paradoxically, by a reinforcement of the ideal of *communitas.* The first happens because city streets have been emptied of crowds and only lonely citizens who work ( Figures 4 and 5) or go shopping ( Figure 2) move through them and, although they form lines ( Figures 2 and 3), they maintain their social distancing, which means that they are autonomous and independent, with their thoughts, their bodies, and their emotions kept to themselves and completely ignoring those of other people. Even the brotherhood members who traditionally share the celebration of the Holy Week are now isolated in their homes, the only trace left of that collective fusion being a black outfit and a flag which helps them to express their inconsolable frustration (7). Patients, for their part, have become more “pathogenic” ( Figures 10 and 11), as they are isolated and, despite being surrounded by several professionals who do their best to look after them and preserve their health, the fragility of their bodies has intensified to such an extent that one leg ( Figure 11) is no longer capable of walking, but to remain prostrate. Consequently, this leg has become a sign of the extreme fragility of the helpless members of the *communitas.* To this it should be highlighted that in the worst case scenario, many patients will die ( Figure 10) a doubly lonely death because, in addition to dying alone, they will pass away without the company of their loved ones. The architecture of dwellings and buildings, which intrinsically encourages fragmentation, segmentation, and social distance ( Figures 8 and 9), can now further strengthen the isolation of its inhabitants, locked inside their homes, separated from one another by corridors, balconies, handrails, openings, and different heights, and also from the street, because they find themselves at a higher level that keeps them far from the daily urban pulse. In short, despite the increased individualism, it takes on a less substantial character and remains closer to the inert townscape objects. In any case, if individualism — and with it the society of individualization and separativity — has seen the birth of new defining aspects, so has the community ideal. Inside homes, mobiles phones and computers ( Figures 8 and 9) have become the only means to communicate with our loved ones, friends, and relatives locked in their homes too. Family members have shared life more than ever before, and neighbours have forced themselves to chat with one another ( Figure 8). In hospitals ( Figures 10 and 11), the bustle, the feverish dynamics, the heart rate, the vital intensity, the effervescent energy, the merging emotions, the empathy, and solidarity have been complemented by the strong will to help others and to leave this situation behind together. Consequently, the ethics of neighbourliness and fraternity to which Max Weber ^51^ referred has been renewed in some of its aspects. However, this ideal of *communitas* arises from absence and emptiness and can therefore not escape a certain void feeling, without forgetting that it has reached a greater pathos which used to characterize the temporary religious ritual, which is now normalized and metamorphosed into daily life.

## Data availability statement

No data are associated with this article.

## References

[1] Beck U : *La sociedad del riesgo global.* Madrid: Siglo XXI;2006.

[2] Beck U Bech-Gernsheim E : La individualización. *El individualismo institucionalizado y sus consecuencias sociales y políticas.* Barcelona: Paidós;2016.

[3] Beck U : *The Metamorphosis of the World: How Climate Change Is Transforming Our Concept of the World.* Cambridge, UK: Polity;2016.

[4] Beck U : Risk Society: Towards a New Modernity. London-NY: SAGE;1992.

[5] Lash S : *Sociología del posmodernismo.* Buenos Aires: Amorrortu;2007.

[6] Bauman Z : *Modernidad líquida.* Buenos Aires: FCE;2003.

[7] Bauman Z : *Vida líquida.* Barcelona: Paidós;2006.

[8] Scheff T : What's love got to do with it? The social-emotional world of pop songs. London-New York: Routledge;2016.

[9] Tönnies F : *Community and Civil Society.* Cambridge: Cambridge University Press;2001.

[10] Weber M : *Basic concepts in Sociology.* New York: Philosophical Library Inc;1962.

[11] González García JM : Sociología e iconología. *REIS, Revista Española de Investigaciones Sociológicas.* 1998;84:23–43.

[12] Echavarren JM : Sociología visual: la construcción de la realidad social a través de la imagen. *Documentos de trabajo (Centro de Estudios Andaluces).* 2010;2(2):1–13.

[13] Davila Legerén A : A la luz de la propia sombra. Incorporaciones de la fotografía a la sociología. *Fotocinema, Revista científica de cine y fotografía.* 2015; (10):285–326.

[14] Bourdieu P : Un art moyen: Essai sur les usages sociaux de la photographie. París: Les Éditions de Minuit;1965.

[15] Goffman E : *Gender Advertisement.* New York: Harper and Row;1979.

[16] Becker HS : *Exploring Society Photographically.* Evanston, Illinois: Mary and Leigh Block Gallery, Northwestern University;1981.

[17] Becker HS : *Art Worlds.* Berkeley: University of California Press;1982.

[18] Bericat Alastuey E : *Sociologías en tiempos de transformación social.* Madrid: Centro de Investigaciones Sociológicas;2012b.

[19] Bericat Alastuey E : Ciencias Sociales y cultura audiovisual: El conocimiento de la fotografía. In: Roche Cárcel JA (Ed.) *La sociología como una de las bellas artes.* Barcelona: Anthropos;2012a;201–224.

[20] Faccioli P Losacco G : *Manuale di sociologia visuale.* Milano: Franco Angeli;2003.

[21] Appadurai A : Mondialisation, Recherche, Imagination. *RISS/ISSJ.* 1999;160:257–268.

[22] Harper D : Visual sociology: Expanding sociological vision. *Am Sociol.* 1988;19(1):54–70. 10.1007/BF02692374

[23] Amezaga BR : Las imágenes como fenómeno cultural: una necesaria mirada en etapas para abordar los retos actuales. *Historia y Memoria de la Educación.* 2019; (10):17–49.

[24] López Del Ramo J Humanes ML : Análisis de contenido de la representación fotográfica de la crisis de los refugiados sirios y su incidencia en el framing visual. *Scire.* 2016;22(2):87–97.

[25] De Andrés S Nos-Aldás E García-Matilla A : La imagen transformadora. El poder de cambio social de una fotografía: la muerte de Aylan. Comunicar. *Revista Científica de Educomunicación.* 2016;47 XXIV:29–37.

[26] Aguilar MJ : Usos y aplicaciones de la Sociología Visual en el ámbito de las migraciones y la construcción de una ciudadanía intercultural. *Tejuelo.* 2011;12:100–135.

[27] De Miguel JM Ponce De Leon O : Para una Sociología de la fotografía. *REIS.* 1998;84(/98):83–124.

[28] Abreu C : El análisis cualitativo de la foto de prensa. *Revista Latinoamericana de Comunicación Social.* 2004;57:1–5.

[29] Muñiz C : Imágenes de la inmigración a través de la fotografía de prensa. Un análisis de contenido. *Comunicación y sociedad.* 2006;XIX(/1):103–128.

[30] Ricoeur P : Hermenéutica y acción. *De la hermenéutica del texto a la hermenéutica de la acción.* Buenos Aires: Prometeo;2008.

[31] Beltrán M : *Dramaturgia y hermenéutica: para entender la realidad social.* Madrid: Centro de Investigaciones Sociológicas;2016.

[32] Grondin J : *A la escucha del sentido: conversaciones con Marc-Antoine Vallée.* Barcelona: Herder;2014.

[33] Van Dijk TA : *Ideología. Una aproximación multidisciplinaria.* Gedisa: Barcelona;1998.

[34] Panofsky E : *Studies in iconology. Humanistic Themes In the Art of the Renaissance.* Boulder-Oxford: Westview Press;1972.

[35] Panofsky E : *Meaning in the Visual Arts.* Chicago: The University of Chicago Press;1983.

[36] Barboza Martínez A : Las imágenes como objeto y técnica de análisis en la Sociología: el método de la interpretación documental.In: Roche Cárcel JA Oliver Narbona M (eds.), *Cultura y globalización.* Entre el conflicto y el diálogo, Alicante: Publicaciones de la Universidad de Alicante;2005;347–366.

[37] Carter MJ : The Hermeneutics of Frames and Framing: An Examination of the Media’s Construction of Reality. *SAGE Open.* 2013. 10.1177/2158244013487915

[38] Lavell A Mansilla E Maskrey A : La construcción social de la pandemia COVID-19: desastre, acumulación de riesgos y políticas públicas. *La Red (Red de Esudios Sociales en Prevención de Desastres en América Latina).* 2020. Reference Source

[39] García-Lastra M : Crisis, pandemia y fragilidades: reflexiones desde un “balcón sociológico”. *Revista de Sociología de la Educación-RASE.* 2020;13(2):140–144.

[40] Pleyers G : *Global Sociology in Times of Coronavirus.* Belgium: Université Catholique de Louvain);2020.

[41] Esquinas MF : Sociología y Ciencias Sociales en tiempos de crisis pandémica. *Revista de Sociología de la Educación-RASE.* 2020;13(2):105–113.

[42] Horkheimer M Adorno T : *Dialectic of enlightenment: philosophical fragments.* Stanford: Stanford University Press;2002.

[43] Augé M : *Pour une anthropologie de la mobilité.* Paris: Payot and Rivages, coll. Manuels Payot;2009.

[44] Alcalá FG : Conjurar el miedo: El concepto Hogar–Mundo derivado de la pandemia COVID-19. *Revista Latinoamericana de Investigación Social.* 2020;3(1):2–26.

[45] Rivero SC : Sociología de las relaciones familiares e intergeneracionales en periodo pandémico. In: Vázquez Atochero A Rivero SC (eds.) *Reflexiones desconfinadas para la era posCOVID-19.* Badajoz: AnthropiQa;2020;105–134.

[46] Pulido CM Villarejo-Carballido B Redondo-Sama G : COVID-19 infodemic: More retweets for science-based information on coronavirus than for false information. *Int Sociol.* 2020;35(4):377–392.

[47] Maderuelo J : *La Idea de Espacio.* Madrid: Akal;2008.

[48] Iglesias RLB Alonso AI : Democracias en cuarentena: respuestas políticas a COVID-19 y el futuro de la democracia. *Revista Española de Sociología.* 2020;29(3):703–714.

[49] Bauman Z Leoncini T : *Generación líquida. Transformaciones en la era 3.0.* Barcelona: Paidós;2018.

[50] Balandier G : *El desorden. La teoría del caos y las ciencias sociales.* Barcelona: GEDISA;2014.

[51] Bellah R : La religión en la evolución humana. *Del Paleolítico a la era axial.* Madrid: CIS;2017.

