# Supplementary material for: The city emptied and the homes and hospitals turned into 'the world'. A sociological approach
Source: F1000Res. 2021 May 27;10:424. [Version 1] doi: 10.12688/f1000research.52097.1 (PMC8822585; doi:10.12688/f1000research.52097.1)
Supplement: Peer review history

# Peer review: Reviewer response for version 1

**DOI:** 10.5256/f1000research.55327.r86291

**Published:** 2021-06-09

**Authors:** Pedro Lisdero

**Affiliations:** 1 CIECS-CONICET, National University of Córdoba, Córdoba, Argentina

Through the analysis of journalistic images, the authors investigate the impact of home confinement around the COVID-19 pandemic in Spain. The "images" are used as an anticipatory formulation of meaning to interpret some relevant signs of social relations in times of pandemic. The article highlights an interesting theoretical-methodological construction that makes it possible to recover the power of "the visual" in the understanding of "society". From some virtuous crossroads between "iconological analysis, the documentary method, and the visual frame", images are understood beyond a mere vehicle for research, to unravel what they show from a non-translatable language about social reality. Thus, the images of Alicante (by photojournalist Rafa Arjones), analysed from their capacity to "show" the meanings constructed around the "Home-World" and the "Hospital-World", constitute a "message" (sensu Melucci) about the re-structuring of the processes of individualism and "communitas" that traverse Spanish society. These tendencies converge in a paradox "visualised" on the basis of pandemic confinement, reinforcing the productivity of "the gaze" proposed by the authors, and opening up questions about the processes of social structuring underway.

Is the work clearly and accurately presented and does it cite the current literature?

Yes

If applicable, is the statistical analysis and its interpretation appropriate?

Not applicable

Are all the source data underlying the results available to ensure full reproducibility?

Yes

Is the study design appropriate and is the work technically sound?

Yes

Are the conclusions drawn adequately supported by the results?

Yes

Are sufficient details of methods and analysis provided to allow replication by others?

Yes

Reviewer Expertise:

Sociology, Visual Sociology, Creative Methodologies.

I confirm that I have read this submission and believe that I have an appropriate level of expertise to confirm that it is of an acceptable scientific standard.

# Peer review: Reviewer response for version 1

**DOI:** 10.5256/f1000research.55327.r86292

**Published:** 2021-06-04

**Authors:** Mauro Guilherme Pinheiro Koury

**Affiliations:** 1 Federal University of Paraíba, João Pessoa, Brazil

The article 'The city emptied and the homes and hospitals turned into 'the world'. A sociological approach' is an instigating study about the daily life lived during an extreme situation, in the COVID-19 worldwide pandemic. Its thematic universe is located in the city of Alicante, Spain, and comprises the changes in the daily lives of a population in the dark times of the pandemic. The authors use visual sociology as a theoretical-methodological resource, in which they figure everyday situations, places of belonging, and conflicting and tense institutional spaces where tomorrow - hope - becomes an unattainable and anguished dream, on the one hand. On the other, an illusioned possibility of a tomorrow, if it arrives, with profound changes in sociability between people and construction of a new - and better - world.

Is the work clearly and accurately presented and does it cite the current literature?

Yes

If applicable, is the statistical analysis and its interpretation appropriate?

Not applicable

Are all the source data underlying the results available to ensure full reproducibility?

Yes

Is the study design appropriate and is the work technically sound?

Yes

Are the conclusions drawn adequately supported by the results?

Yes

Are sufficient details of methods and analysis provided to allow replication by others?

Yes

Reviewer Expertise:

Anthropology of Emotions; Urban Anthropology; Anthropological Theory; Visual Anthropology

I confirm that I have read this submission and believe that I have an appropriate level of expertise to confirm that it is of an acceptable scientific standard.

# Peer review: Reviewer response for version 1

**DOI:** 10.5256/f1000research.55327.r86294

**Published:** 2021-06-01

**Authors:** Francesca Randazzo Eisemann

**Affiliations:** 1 National Autonomous University of Honduras, Tegucigalpa, Honduras

The sociological approach is enriched by iconological analysis and the visual frame. The authors put into value the content of the photographs that mark a concrete experience related to COVID pandemics. It is both an important academic and ethnographic testimony.

- The literature cited corresponds to in vogue sociology theoreticians and visual analysis.
- The design is adapted to the scientific article model.
- Qualitative data is provided and illustrates the purpose of the article.
- One of the most interesting conclusions is the disruption of the social order translated into geometrical terms, specifically the collapse of coordinates and axis of social life.

Is the work clearly and accurately presented and does it cite the current literature?

Yes

If applicable, is the statistical analysis and its interpretation appropriate?

Not applicable

Are all the source data underlying the results available to ensure full reproducibility?

Yes

Is the study design appropriate and is the work technically sound?

Yes

Are the conclusions drawn adequately supported by the results?

Yes

Are sufficient details of methods and analysis provided to allow replication by others?

Yes

Reviewer Expertise:

Social Imaginaries, Qualitative research, Gender, Microsociology, Garifunas

I confirm that I have read this submission and believe that I have an appropriate level of expertise to confirm that it is of an acceptable scientific standard.
